# Dendritic Branching of Olfactory Bulb Mitral and Tufted Cells: Regulation by TrkB

**DOI:** 10.1371/journal.pone.0006729

**Published:** 2009-08-25

**Authors:** Fumiaki Imamura, Charles A. Greer

**Affiliations:** 1 Department of Neurosurgery, Yale University School of Medicine, New Haven, Connecticut, United States of America; 2 Department of Neurobiology, Yale University School of Medicine, New Haven, Connecticut, United States of America; Duke Unviersity, United States of America

## Abstract

**Background:**

Projection neurons of mammalian olfactory bulb (OB), mitral and tufted cells, have dendrites whose morphologies are specifically differentiated for efficient odor information processing. The apical dendrite extends radially and arborizes in single glomerulus where it receives primary input from olfactory sensory neurons that express the same odor receptor. The lateral dendrites extend horizontally in the external plexiform layer and make reciprocal dendrodendritic synapses with granule cells, which moderate mitral/tufted cell activity. The molecular mechanisms regulating dendritic development of mitral/tufted cells is one of the unsolved important problems in the olfactory system. Here, we focused on TrkB receptors to test the hypothesis that neurotrophin-mediate mechanisms contributed to dendritic differentiation of OB mitral/tufted cells.

**Principal Findings:**

With immunohistochemical analysis, we found that the TrkB neurotrophin receptor is expressed by both apical and lateral dendrites of mitral/tufted cells and that expression is evident during the early postnatal days when these dendrites exhibit their most robust growth and differentiation. To examine the effect of TrkB activation on mitral/tufted cell dendritic development, we cultured OB neurons. When BDNF or NT4 were introduced into the cultures, there was a significant increase in the number of primary neurites and branching points among the mitral/tufted cells. Moreover, BDNF facilitated filopodial extension along the neurites of mitral/tufted cells.

**Significance:**

In this report, we show for the first time that TrkB activation stimulates the dendritic branching of mitral/tufted cells in developing OB. This suggests that arborization of the apical dendrite in a glomerulus is under the tight regulation of TrkB activation.

## Introduction

Projection neurons in the olfactory bulb (OB), mitral and tufted cells, receive olfactory information from olfactory sensory neurons (OSNs) and process the signal within the OB before sending it to the olfactory cortices. To perform these functions efficiently, they have two types of dendrites that are morphologically and functionally distinct; the apical and lateral dendrites. In adult OB, mitral/tufted cells extend radially a single apical dendrite that arborizes in a complex tuft within one glomerulus, where it receives OSN synaptic input. In contrast, each mitral/tufted cell extends several lateral dendrites that are widely distributed within a horizontal plane in the external plexiform layer (EPL), and make reciprocal dendrodendritic synapses with granule cells.

During development, mitral cells possess multiple widely spread dendrites that cannot be readily classified as apical or lateral on the day of birth; only a few cells show arborized terminal dendritic tufts [1]–[3]. Within a few days, evidence of differentiation can be detected as one apical dendrite appears thicker. However, as late as postnatal day 5 (P5) many mitral cells continue to have multiple apical dendrites, some of which project into different glomeruli. By P10, the supernumerary apical dendrites have retracted from glomeruli, and most mitral/tufted cells have adult-like morphology with only a single apical dendrite. While in the mature rodent the lateral dendrites of mitral and tufted cells segregate in the deep and superficial substrata of the EPL, respectively, little is known about the time course or mechanisms underlying their development [4], [5].

What regulates the dynamic, but organized, developmental changes of mitral/tufted cell dendritic morphology? Signaling via TrkB neurotrophin receptor is a good candidate since activation of TrkB via its ligands, brain-derived neurotrophic factor (BDNF) or neurotrophin-4 (NT4), can stimulate formation of apical dendrites and branching in cortical pyramidal neurons [6]–[8]. However, the function of TrkB appears to differ among different types of neurons. In the retina, specific TrkB deletion in the ganglion cells retards the laminar refinement of dendrites [9]. In contrast, TrkB deletion in cerebellar Purkinje cells did not affect the gross dendritic morphology, but synapse formation and elimination were affected [10], [11]. Therefore, although TrkB expression in dendrites of mature mitral cells in the OB has been shown [12], an understanding of its role and the implications for dendritic development have been lacking. Here, we report on the localization of TrkB in dendrites of mitral/tufted cells at the peak of dendritogenesis, during the early perinatal period. Our data further show that the addition of BDNF or NT4 to mitral/tufted cells *in vitro* results in a proliferation of primary neurites, neurite branching, and filopodia-like elaboration. Our results strongly support the notion that the selective effects of TrkB activation contribute to the development and differentiation of OB mitral/tufted cell dendrites.

## Results

### Development of dendrites of mitral cells during early postnatal days

**Figure 1:**
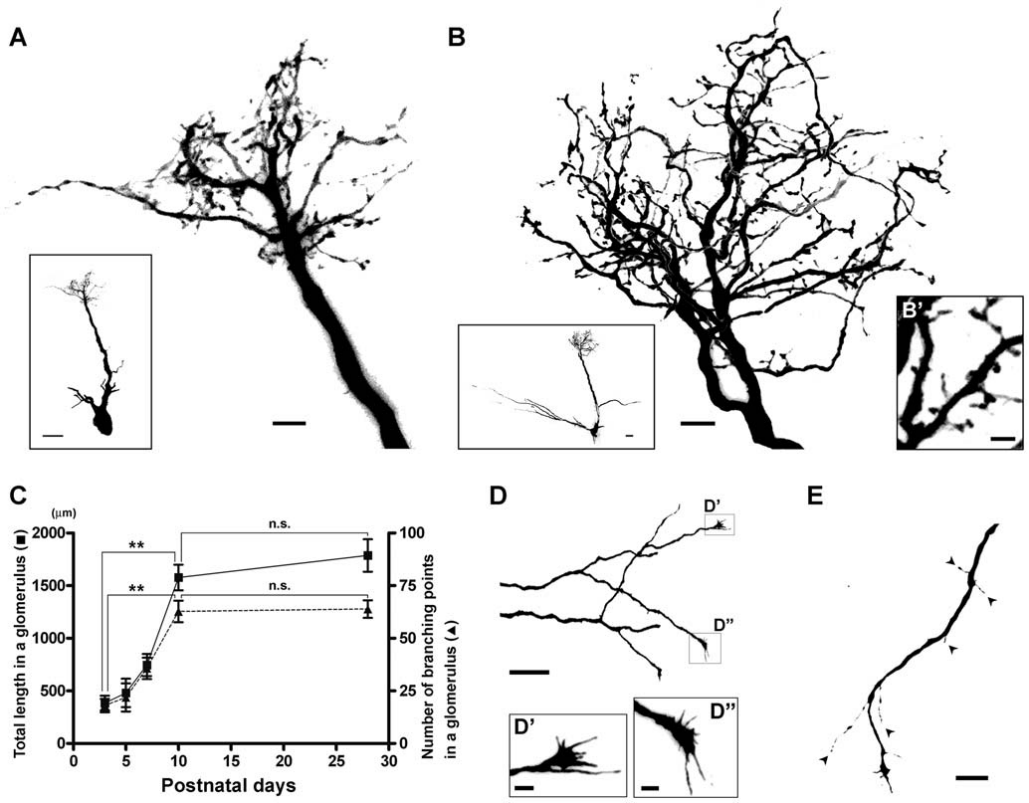
Dendritic morphology of mitral cells. A, B: Lucifer yellow-labeled mitral cell at P3 (A) and P28 (B). Reconstructed cells with whole apical dendrite (insets) and their glomerular tufts are shown. High-magnification view of glomerular tuft at P28 is shown in B'. Many spine-like structures are visible. C: Total dendrite length (squares) and number of branching point (triangles) in a glomerulus were quantified at P3, 5, 7, 10 and 28. Asterisks denote statistically significant differences in total dendrite length and number of branching point between P3 and P10. Unpaired t test was used for statistics (**p<0.0001). Neither total dendrite length nor branching point number are significantly different between P10 and 28 (n.s.). D: Lateral dendrite of mitral cell labeled with Lucifer yellow at P7. Growth cones which have filopodial structures are seen at the tips of dendrites (D', D″). E: Another lateral dendrite labeled at P7. At this age, filopodia (arrowheads) are observed along the dendrite. Scale bars: 20 µm in insets of A and B, D, E; 5 µm in A, B; 2 µm in B', D', D″.

To investigate the dendritic development of mitral cells, we used intracellular injections of Lucifer yellow dye in formaldehyde-fixed slices of the OB from mice at P3, 5, 7, 10 and 28. In Figure 1A and B, we show the reconstructed dendritic tufts of typical dye-injected mitral cells from mice at P3 and P28, respectively. At P3, the apical dendrite of the mitral cell has begun to elaborate an arborized tuft (Fig. 1A). At this age, the apical dendritic specializations still appeared relatively simple. The processes often had many short branches, perhaps indicative of ongoing development and branching. At P28, the dendritic processes of glomerular tufts of mitral cells were tightly intermingled (Fig. 1B). They were indistinguishable from dendritic tufts seen in adult rat OB [13], [14]. In addition to primary, secondary and tertiary branches, numerous smaller filopodia- or spine-like structures were now apparent at P28 (Fig. 1B').

To examine the growth of tufts, we measured the total length and number of branching points of dendrites in glomeruli at each age. At younger ages, mitral cells often had two or more apical dendrites, each of which exhibited an immature tuft in different glomeruli (Fig. S1). In those cases, each tuft was treated as an independent event. Although the filopodia- or spine-like structures noted above are likely important in the maturation of the apical glomerular dendritic tufts, branches shorter than 6 µm were omitted from our measurement since previous report suggested that processes less than 6 µm are less stable, even in adult mitral cell dendrites [15]. The total length of dendritic processes in a glomerulus from single mitral cells increased significantly from P3 to P10, while the change from P10 to P28 was not significant (P3 = 384.1±70.2 µm; P5 = 480.5±134.6 µm; P7 = 745.8±104.8 µm; P10 = 1526.8±121.6 µm; and P28 = 1787.1±154.3 µm; Fig. 1C). Similarly, the number of branching points increased significantly from P3 to P10, but was thereafter relatively stable (P3 = 18±3; P5 = 22±7; P7 = 36±5; P10 = 63±5; and P28 = 64±4; Fig. 1C). These results suggest that early postnatal days, such as P3-10, may be a critical period for the development of mitral cell apical dendritic tufts, and that after P10 a relatively stable equilibrium is achieved.

The lateral dendrites of mitral cells extend up to 1/3 the circumference of the OB, often extending 1,000 µm in length in the rat [5] and 2,000 µm in the rabbit [4]. Because of the length of the lateral dendrites, and because their planes of orientation within the EPL are complicated, it was not feasible to reconstruct them from our slice preparations as we did for the apical dendrites. However, as an alternative measure of maturation, we found that the lateral dendrites exhibited growth cone-like structures at their tips at P3, 5, and 7 (Figs. 1D, D', and D″). This growth cone-like structure was never seen beyond P10. In addition, prior to P10 the lateral dendrites also had a high incidence of filopodia-like extensions which we rarely found in adult OB (Fig. 1E; arrowheads). These data suggest that, like the apical processes, the lateral dendrites are experiencing their most robust growth and development up through P10, with perhaps only incremental changes after that age.

### TrkB expression at dendrites of mitral/tufted cells

**Figure 2:**
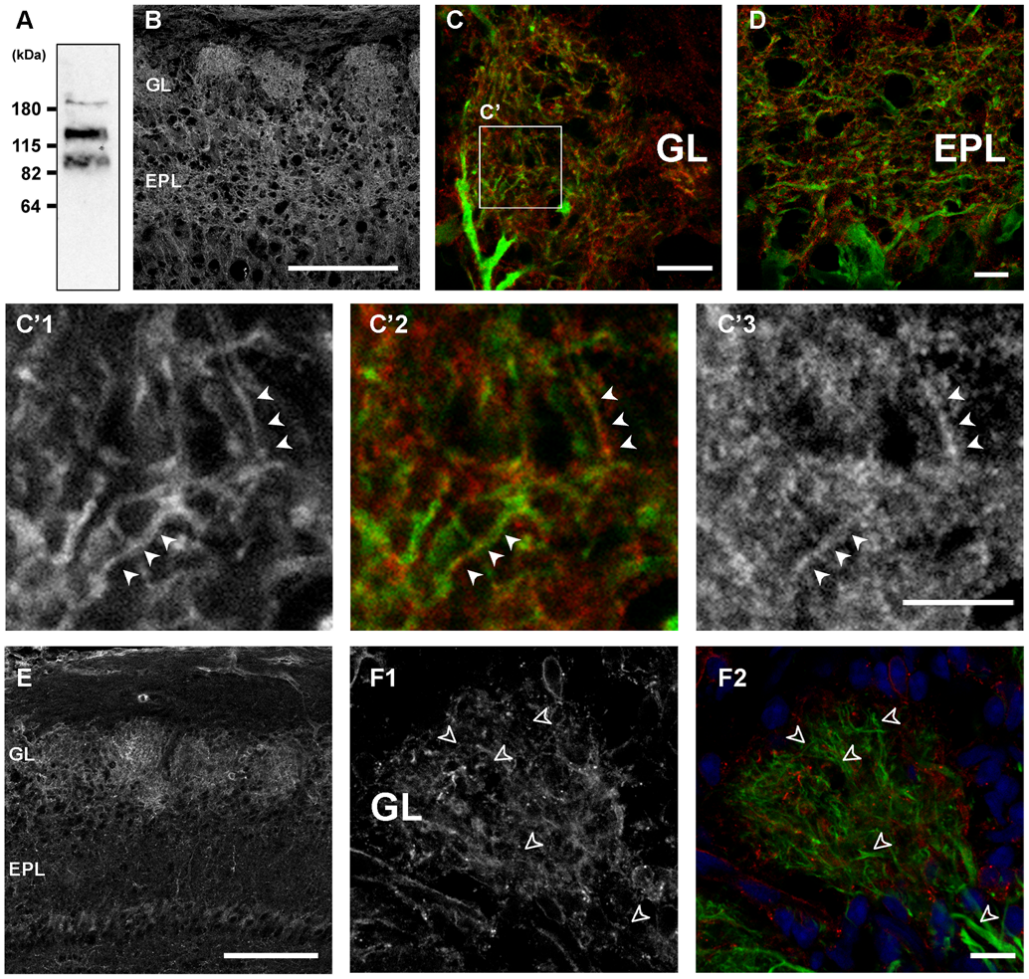
Expression patterns of TrkB and p75NTR in developing OB. A: Western blot analysis with P5 OB homogenate using anti-TrkBex antibody. About 25 µg of protein were loaded. B: P4 OB stained with anti-TrkBex antibody. Signals are observed in GL and EPL. C, D: High magnification views of GL (C) and EPL (D) stained with anti-TrkBex antibody (red) at P4. Dot-like TrkB signals (red) are apposed to both apical and lateral dendrites of mitral/tufted cells (green). C': Higher magnified images from the region encircled in C. TrkB signals (C'3; red in C'2) are positioned on YFP-positive processes (C'1; green in C'2) (arrowheads). E: P7 OB stained with anti-p75NTR antibody. Signals are seen in GL and MCL. F: High magnification views of a glomerulus stained with anti-p75NTR antibody (F1; red in F2). No p75NTR signal is seen at dendrites of mitral/tufted cells (green) (open arrowheads). The thy1-YFP-G mice are used to visualize dendrites of mitral/tufted cells in C, D, and F. Scale bars: 100 µm in B, E; 10 µm in C, D, F; 5 µm in C'.

Previously, it was reported that at P0, *trkB* mRNA is expressed by cells in the mitral cell layer (MCL) as well as in the glomerular layer (GL) and granule cell layer [16], [17]. However, the majority of cells within the MCL are not mitral cells; granule cells are densely packed within the MCL [18]. Moreover, while localization of the mRNA is important, the expression pattern of TrkB protein was not known for those stages of early development when dendritogenesis is most robust. To address this question, we examined TrkB expression using anti-TrkB antibody: anti-TrkBex. Its target sequences are located at extracellular regions of TrkB. There are, at least, three TrkB isoforms that are produced by alternative splicing [19], [20]. The full-length isoform (TrkB.FL) has a catalytic tyrosine kinase domain in its intracellular region. In contrast, the two truncated isoforms (TrkB.T1 and T2) have the same extracellular domains as TrkB.FL, but lack the intracellular tyrosine kinase domains which are substituted with isoform-specific shorter C-terminal sequences. Therefore, anti-TrkBex can detect all TrkB isoforms. To check the expression of TrkB isoforms in neonatal OB, western blots were performed with P5 OB homogenate using anti-TrkBex (Fig. 2A). Bands around 95 kDa and 145 kDa correspond to the molecular weights of truncated and full-length isoforms, respectively. This antibody also detected a band around 180 kDa, which is supposed to be highly phosphorylated form of TrkB [21]. The result shows that both full-length and truncated TrkBs are expressed in the neonatal OB.

When P4 OB slices were stained with anti-TrkBex, strong signals were observed in the GL and EPL (Fig. 2B). To examine TrkB localization in relation to dendrites of mitral/tufted cells, we used the thy1-YFP-G mice in which subsets of mitral/tufted cells express YFP [22]. Apical and lateral dendrites of mitral/tufted cells were clearly observed with YFP signals in GL (Fig. 2C) and EPL (Fig. 2D), respectively. Both in GL and EPL, punctate TrkB signals closely apposed to YFP-positive dendrites were observed. With a high magnification view in a glomerulus (Fig. 2C'), these punctate signals were positioned on YFP-positive processes, which suggests that TrkB signals occur in the mitral/tufted cell dendrites.

Our results suggest that TrkB and its ligands, BDNF and NT4, are involved in dendritic development of mitral/tufted cells. Since the p75 neurotrophin receptor (p75NTR) binds BDNF and NT4 with low affinity and may regulate dendritic development [23], we also examined the p75NTR expression in the early postnatal OB. Here, we observed p75NTR expression in glomeruli at P7 (Fig. 2E). However, no p75NTR signals were colocalized with the YFP seen in mitral/tufted cell dendrites (Fig. 2F). This is consistent with previous reports which showed that p75NTR was shown to be expressed in glomeruli [24] as well as olfactory ensheathing cells [25], but not in mitral cells.

### TrkB activation increased the number of mitral/tufted cells in vitro

**Figure 3:**
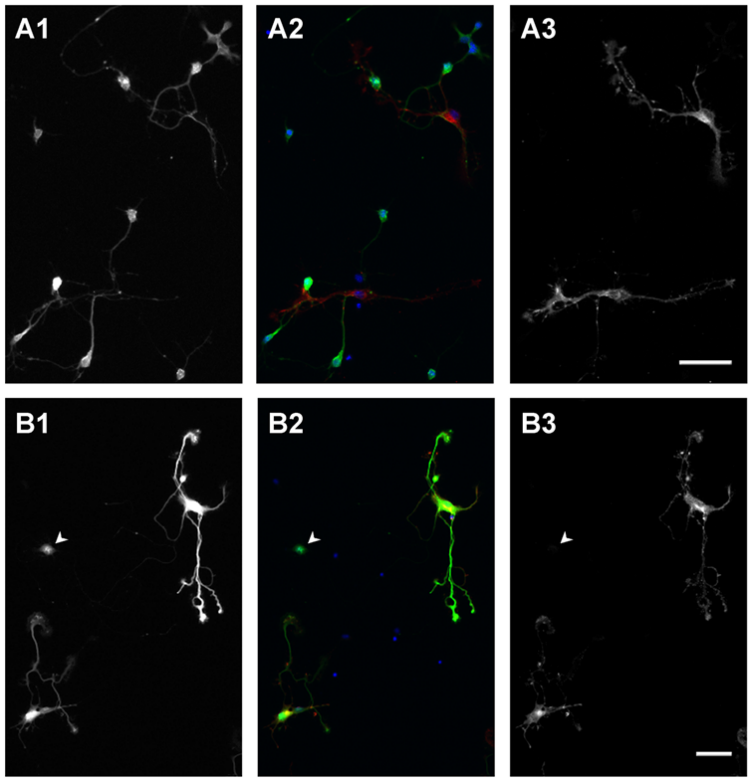
Properties of OB neurons in culture. A: OB neurons cultured from P1 GAD67-GFP mice. At 4 DIV, cells were doubly stained with anti-GFP antibody (A1; green in A2) and anti-vGluT1 antibody (A3; red in A2). No GFP-positive cells express vGluT1. B: OB neurons cultured from P1 thy1-YFP-G mice. At 4 DIV, cells were doubly stained with anti-GFP (YFP) antibody (B1; green in B2) and anti-vGluT1 antibody (B3; red in B2). Many YFP-positive cells coexpress vGluT1. YFP-positive but vGluT1-negative cell also exists in culture (arrowhead). Scale bars: 50 µm.

We next asked if activation of TrkB receptors affected the development of mitral/tufted cells, using primary cultures of dissociated OB neurons. We had several strategies that enabled us to specifically identify mitral/tufted cells *in vitro*. The vesicular glutamate transporter 1 (vGluT1) is a membrane protein associated with glutamate accumulation in vesicles, and, therefore, is known as a marker for excitatory neurons. In the OB, vGluT1 is expressed only in mitral/tufted cells, and it is not found in granule or periglomerular cells [26]. As expected, when OB neurons were cultured from GAD67-GFP knockin mice, in which GFP is expressed by granule and periglomerular cells [27], [28], no vGluT1 expression was observed in GFP positive cells (Fig. 3A). In contrast, YFP-positive cells dissociated from the thy1-YFP-G mice coexpressed vGluT1 at 4 days *in vitro* (DIV) (Fig. 3B). There were also some vGluT1-negative YFP-positive cells (Fig. 3B arrowhead) that are most likely immature cells. Hereafter, we considered YFP-positive cells as representative of mitral/tufted cells *in vitro*.

**Figure 4:**
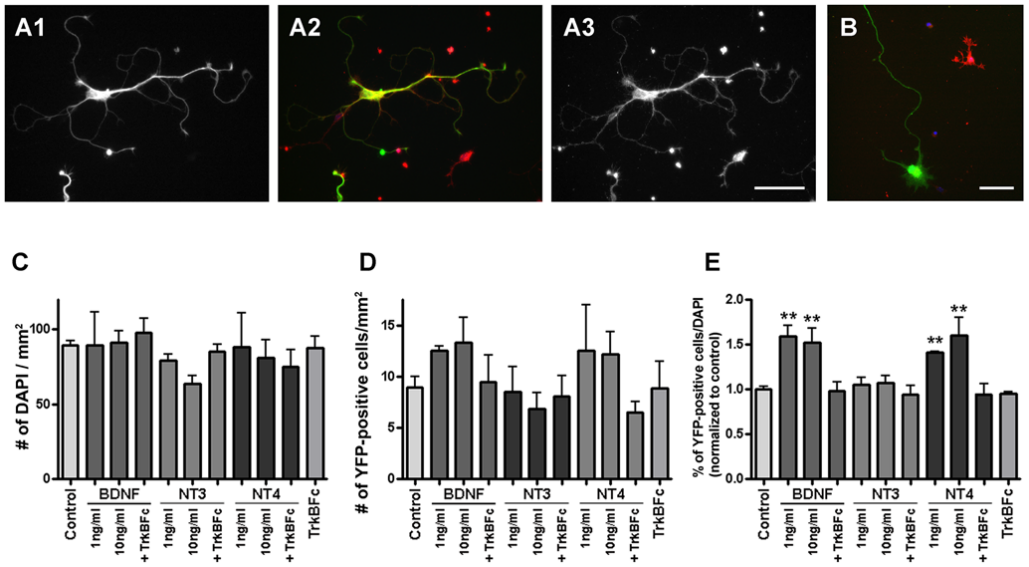
Increase of YFP-positive cells by TrkB activation. A: OB neurons cultured from P1 thy1-YFP-G mice were doubly stained with anti-YFP antibody (A1; green in A2) and anti-TrkBex antibody (A3; red in A2) at 2 DIV. All YFP-positive cells coexpress TrkB. B: The p75NTR (red) is expressed by fibroblast-like cells and not by YFP-positive cells (green). Scale bars: 50 µm. C–E: Densities of DAPI (C) and YFP-positive cells (D) are measured after being cultured for 4 days with/without neurotrophins, and percentages of YFP-positive cells among DAPI are calculated and graphed after normalizing to control (E). BDNF, NT3, or NT4 were added to medium with final concentration of 1 or 10 ng/ml. When TrkBFc was applied, it was added to medium at a final concentration of 1 µg/ml with 10 ng/ml of neurotrophins. Application of BDNF or NT4 increases the proportion of YFP-positive cells in culture. Every condition was tested at least three times. Asterisks in E denote statistically significant differences from control condition analyzed with unpaired t test (**p<0.0001).

Staining with anti-TrkBex antibody, we found that most cells including YFP-positive cells expressed TrkB at 2 DIV (Fig. 4A). In YFP-positive cells, TrkB was evenly distributed in the cell and its processes. Consistent with the *in vivo* expression pattern, no YFP-positive cells expressed p75NTR. Only fibroblast-like cells were p75NTR-positive at this time point (Fig. 4B). To activate TrkB on the cells, we applied neurotrophins, BDNF, NT3, or NT4, in the culture medium from 0 to 4 DIV. At 4 DIV, cells were fixed and immunostained with anti-GFP antibody to enhance the YFP signals for analysis. First, we measured the number/density of DAPI-positive (Fig. 4C) and YFP-positive cells (Fig. 4D) under our culture conditions. In order to exclude the dying cells, YFP-positive cells that did not have a neurite longer than 10 µm were not counted in this analysis. The percentages of YFP-positive cells among the DAPI-positive cells in different conditions are normalized to control in Figure 4E. We found that application of BDNF or NT4 increased the percentages of YFP-positive cells in culture about 1.5-fold, while NT3 had no significant effect on cell density. Because both NT4 and BDNF have the highest affinity for TrkB, while NT3 has a lesser affinity [29], these data strongly indicate that the increase in the number of YFP-positive cells is most likely mediated via TrkB activation. To test this further, we added to the culture medium the chimeric protein of human TrkB and Fc (TrkBFc; 1 µg/ml). Under these conditions the effects of BDNF or NT4 were quenched.

Because the density of DAPI-positive nuclei was not significantly different among conditions (Fig. 4C), the change in the percentage of YFP-positive cells among DAPI-positive cells caused by BDNF or NT4 application has to be due to increase of YFP-positive cells in culture. The most plausible explanation is that survival of YFP-positive cells was promoted by TrkB activation as has been shown for several types of neurons [30]. Another possibility is that TrkB activation induced YFP expression in cells which were originally YFP-negative.

### TrkB activation induced new branch and primary neurite formation

**Figure 5:**
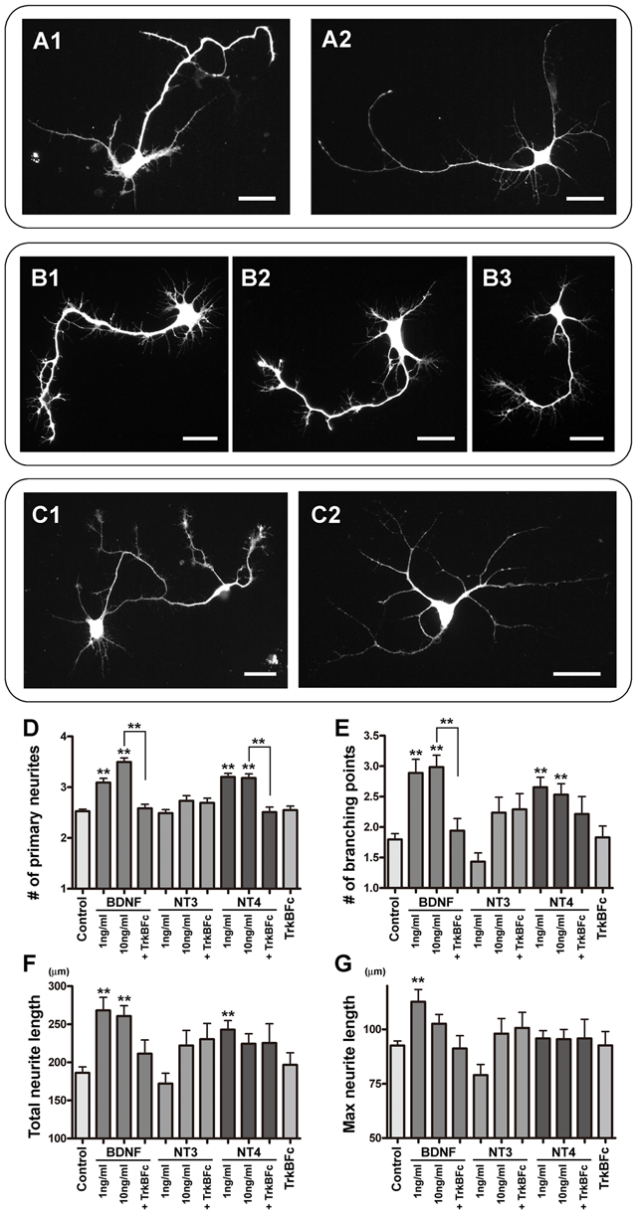
Stimulation of neurite development by TrkB activation. A–C: Representative YFP-positive cells cultured from P1 thy1-YFP-G mice. The cells were cultured for 4 days in control medium (A), with 10 ng/ml of BDNF (B), or with 10 ng/ml BDNF and 1 µg/ml of TrkBFc (C). YFP signals were enhanced with anti-GFP antibody. More short branches and primary neurites are formed in the cells treated with BDNF compared to control. D–G: YFP-positive cells were cultured with/without neurotrophins for 4 days as described in figure 4. Numbers of primary neurites (D) and branching points (E), total neurite length (E), and maximum neurite length (F) of YFP-positive cells with different culture conditions are measured and averaged. At least 76 YFP-positive cells were analyzed for each condition and the experiments were repeated three times. Asterisks denote statistically significant differences from control condition or differences between two conditions linked with a bar. Unpaired t tests were used for statistics (**p<0.0001). Scale bars: 25 µm.

Next, we examined the effects of TrkB activation on neurite development of YFP-positive cells. At 4 DIV, under control conditions many YFP-positive cells had several primary neurites that extended from the cell body, some with branches (Fig. 5A). When 10 ng/ml of BDNF was added to the medium from 0 to 4 DIV, we found many short branches along neurites of YFP-positive cells, although the gross morphologies were not dramatically different when compared to control cells (Fig. 5B). In addition to the increased number of short branches, the number of primary neurites which emerge from the cell body also increased following BDNF treatment. When the BDNF in medium was quenched with the addition of 1 µg/ml of TrkBFc chimera proteins, the frequency of short branches did not change relative to controls (Fig. 5C).

To quantify the effects of neurotrophins on morphology, the mean numbers of primary neurites (Fig. 5D) and branching points (Fig. 5E) were measured from YFP-positive cells. Here, we analyzed the morphology of YFP-positive cells using the images taken with 10×objective. Since the minimum resolution of this lens is 1.3 µm, thin processes, such as filopodia whose diameter are usually 0.2–0.6 µm, were not taken into account in the total neurite length or number of branching points shown in Figures 5F and 5E, respectively (Fig. S2). Neurites or branches less than 10 µm were also excluded from this analysis. We measured a minimum of 76 YFP-positive cells per condition, and each experiment was repeated three times. As shown in Figures 5D and E, both the numbers of primary neurites and branches were significantly increased with BDNF or NT4 application. As above, the addition of TrkBFc diminished the effects of BDNF and NT4. These effects also seem most likely to be mediated via TrkB activation since NT3 did not result in any measurable changes in these parameters. Total neurite length was also increased by BDNF or NT4 (Fig. 5F). This was largely due to increase of primary neurites and branches since the maximum neurite length was not markedly different (Fig. 5G). Therefore, TrkB activation appears important for initiating new primary neurites or branches in YFP-positive cells, but may not contribute to the elongation of the pre-existing neurites.

**Figure 6:**
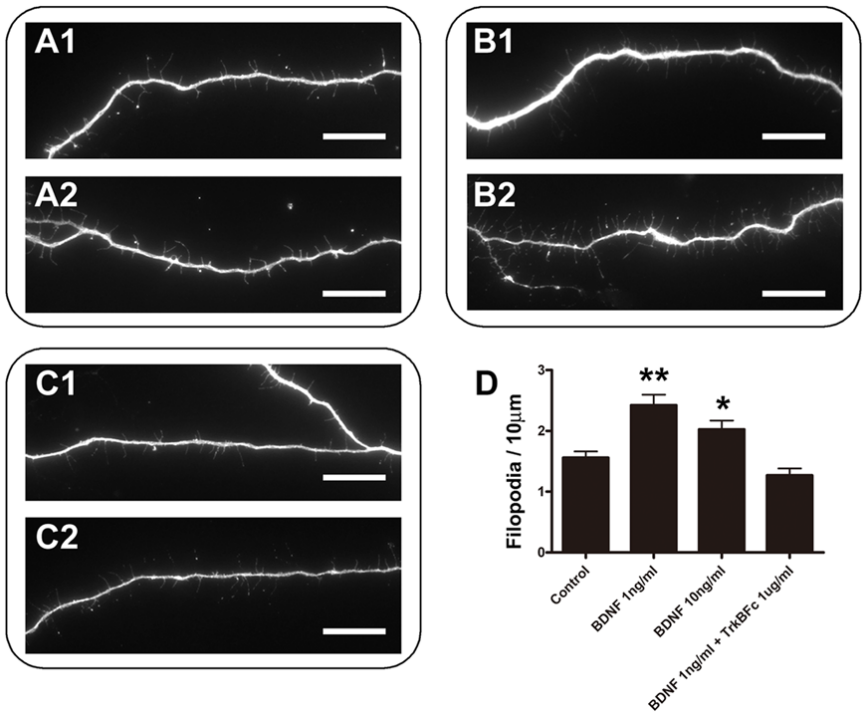
Effects of TrkB activation on filopodia growth. A–C: Filopodia of YFP-positive cells cultured from P1 thy1-YFP-G mice. Cells cultured for 4 days in control medium (A), with 1 ng/ml of BDNF (B), or with 1 ng/ml BDNF and 1 µg/ml of TrkBFc (C) are shown. D: Filopodia densities at each indicated condition were measured. The experiments were repeated three times, and in total 78, 41, 42, and 39 cells were analyzed for control, 1 ng/ml BDNF, 10 ng/ml BDNF, and 1 ng/ml BDNF and 1 µg/ml TrkBFc, respectively. Asterisks denote statistically significant differences from control condition or differences between two conditions linked with a bar. Unpaired t tests were used for statistics (**p<0.001, *p<0.05). Scale bars: 20 µm.

At higher magnifications, taken with 60×objective, it was apparent that the YFP-positive cells had short filopodia distributed along their neurites at 4 DIV (Fig. 6A and Fig. S2). Because we also observed filopodia on growing lateral dendrites of mitral/tufted cells *in vivo* (Fig. 1E), we next examined the effects of BDNF on filopodia which are thin and short protrusions extending from thicker neurites. Application of 1 ng/ml of BDNF to the culture medium caused a marked change in the filopodia extending from the neurites of YFP-positive cells (Fig. 6B). Density and length were both increased. We measured the densities of filopodia at each condition, as shown in Figure 6D. We should note that since we defined all thin protrusions as filopodia, some filopodia longer than 10 µm might be defined also as branches in the analysis shown in Figure 5B. The densities of filopodia were significantly increased with BDNF application: 2.4±0.2 (*n* = 41 cells) or 2.0±0.1 (*n* = 42 cells) filopodia/10 µm was observed when 1 or 10 ng/ml BDNF was applied, respectively. This contrasts with a value of 1.6±0.1 filopodia/10 µm (*n* = 78 cells) seen in control cells (Fig. 6D). The BDNF induced increase in filopodial density disappeared when TrkBFc was added to medium: 1.3±0.1 filopodia/10 µm (*n* = 39 cells) (Figs. 6C and D). It was previously shown that dendritic filopodia can be precursors for new branches [31] as well as spines [32]. Therefore, taken together, our results suggest that TrkB activation is important for dendritic development of mitral/tufted cells and likely stimulates the growth of filopodia, leading to the formation of new neurites, branches, and spines.

## Discussion

### Role of TrkB in dendritic development of mitral/tufted cells

In this paper, we show, for the first time, the involvement of TrkB in dendritic development of mitral/tufted cells in the OB. We found that TrkB is expressed at both apical and lateral dendrites of mitral/tufted cells during early postnatal development, when apical and lateral dendrites grow and dramatically change their morphologies. We also found that *in vitro* TrkB activation with BDNF or NT4 stimulated the formation of new neurite branches in YFP-positive cells, mitral/tufted cells, with less effect on the elongation of neurites. These data are consistent with cortical neuron dendritic branch formation, both *in vivo* and *in vitro* [7], [8]. Consequently, we suggest that TrkB activation plays an important role in the formation of new dendritic branches on mitral and tufted cells.

In the adult, the apical dendrites of mitral/tufted cells are branchless as it course through the EPL, and arborize after they reach the glomerular neuropil. It was shown that the olfactory epithelium influences the orientation [33] and elongation, but not branching [34], of mitral/tufted cell dendrites during prenatal development. The TGF-beta superfamily proteins are suggested to promote dendritic elongation [34]. TrkB on apical dendrites may be activated only after they begin to innervate a glomerulus. As we discuss later, local interactions, perhaps with OSN axons, are necessary for TrkB activation, which leads to new branch formation in apical dendrites of mitral/tufted cells. Although little is known about the development of lateral dendrites of mitral/tufted cells in the EPL, we did find filopodia along growing lateral dendrites. Moreover, we report here that TrkB activation increased filopodia density along YFP-positive cells. Since filopodia are known as precursors for new branches [31], it is plausible that TrkB is also involved in branching of lateral dendrites in the EPL.

The expression of BDNF in the mouse olfactory system remains controversial. Cao et al. (2007) used immunohistochemistry to show BDNF expression in mouse OB glomeruli at P14, but did not achieve a definitive cellular localization [35]. Because BDNF can be transported to both axon terminals and dendrites in neurons [36], both OSNs and cells in the OB are possible sources for the release of BDNF. Feron et al. (2008) reported on BDNF expression in OSNs with immunohistochemistry and Clevenger et al. (2008) found BDNF promoter driven β-galactosidase expression in a small subset of OSNs [37], [38]. Others, however, report the absence of BDNF mRNA and protein in OSNs [17], [35]. BDNF expression in the OB is also inconsistent. Whereas some reported BDNF expression in mitral cells [35], [39], others did not find BDNF-positive mitral cells [17], [37]. The periglomerular cells are another candidate for the BDNF source in glomeruli [35], [37], although the genesis and arrival of periglomerular cells occurs rather late in development to mediate the effects we report here [40]–[42]. Moreover, there are no reports of NT4 expression in the olfactory system, or expression of neurotrophins in the EPL. Therefore, further detailed analyses of neurotrophin expression in the mouse olfactory system during early postnatal days are required, although the data we report here make a compelling case for the role of TrkB.

### Critical period for dendritic development of mitral/tufted cells

We showed that the apical dendritic tuft of mitral cells increases in length and number of branching points in glomeruli during early postnatal days, acquiring an adult-like morphology by P10. Once matured, dendritic tufts of mitral cells are less dynamic [15]. This time course is well correlated with an increase in the number of synapses in the GL [43]; synapses are first found at ∼E15 and the density of synapses reaches a peak around P15. *In vivo* imaging in the developing zebrafish showed that synapse formation and dendritic growth occur simultaneously; filopodia which have nascent synapses are stabilized, and stabilized filopodia mature into new branches [31]. From our data, it appears that a comparable developmental strategy may be applicable to dendritic tuft growth of mitral/tufted cells in mouse OB.

Whether new branch formation on mitral/tufted cell dendrites is activity-dependent or not is an important question. We previously showed that the interaction between OSN axons and mitral/tufted cell dendrites to form glomeruli occurs shortly before birth [25]. Functionally defined connections between OSN axons and mitral cells were confirmed at P5 with calcium imaging in mitral cell dendrites after olfactory nerve stimulation [44]. It is clear that BDNF can be released both synaptically and extrasynaptically, and that depolarization and intracellular Ca^2+^ elevation are necessary for BDNF secretion [45]. Thus, it is reasonable to suggest that neuronal activity may be a prerequisite for activation of TrkBs on the dendrites of mitral/tufted cells, which in turn would stimulate new branch formation. Consistent with this idea, mitral cells do not form elaborate tufts at the tips of apical dendrites when OSN axons are ablated from glomeruli by surgery or genetic engineering [46], [47]. However, deletion of the functional CNG channel in OSNs or neonatal naris closure slightly delayed development in apical dendrite of mitral cells, but did not have a profound effect [2], [48]. These latter findings suggest that spontaneous OSN activity may be sufficient to release BDNF from OSN axons and/or mitral/tufted cell dendrites leading to an activation of TrkB.

Our finding that TrkBs regulate dendritic branching of developing OB mitral/tufted cells is the first step toward understanding dendritic differentiation and pruning among these projection neurons. Given the specificity of glomerular innervation by mitral/tufted cell apical dendrites, and the elaborate distribution of the lateral dendrites, it seems likely that complementary mechanisms may emerge that differentially affect apical versus lateral dendrites. As a next step, future studies include analyses of the down-stream signaling of TrkB activation.

## Materials and Methods

### Animals

All the experiments were performed in mice. Unless otherwise noted, we used the CD-1 mouse strain (Charles River Laboratories; Wilmington, MA) as wild-type mice. The thy1-YFP-G mice were kindly provided by Dr. Feng at Duke University [22]. GAD67-GFP knock-in mice [27] were a kind gift from Dr. W. Chen (The University of Texas Medical School at Houston). Genotypes of mice were confirmed by PCR. All animal care and use was approved by the Yale University Animal Care and Use Committee.

### Immunohistochemistry

P4 and P7 mice were rapidly decapitated and immersion fixed in 4% paraformaldehyde (PFA) in phosphate-buffered saline [PBS: 0.1 M phosphate buffer (PB) and 0.9% NaCl (pH 7.4)] at 4°C overnight. Fixed tissues were cryo-preserved in 30% sucrose in 0.1 M PB (pH 7.4), and embedded in O.C.T. compound (Sakura Finetek; Torrance, CA). The olfactory tissues were cut on a cryostat (Reichert-Jung 2800 Frigocut E cryostat) into 20 µm slices, and stored at −20°C until use.

On the day of immunohistochemistry, the slices were first rinsed with TBS-T [10 mM Tris-HCl (pH 7.4), 100 mM NaCl with 0.3% Triton-X], blocked with 3% bovine serum albumin (BSA) and 5% normal donkey serum in TBS-T (blocking buffer) at room temperature for 60 minutes and incubated with primary antibodies diluted in blocking buffer overnight at room temperature. Sections were washed with TBS-T, then incubated with secondary antibodies with 4′,6-diamino-2-phenylindole dihydrochrolide (DAPI) (Invitrogen; Carlsbad, CA), and DRAQ5 (Biostatus Ltd.; Leicestershire, United Kingdom) for nuclei staining for 60 minutes at room temperature. The immunoreacted sections were washed, mounted with Gel/Mount mounting medium (Biomeda; Foster City, CA), and imaged with a laser scanning confocal microscope (Leica TCS SL, Leica Microsystems; Wetzlar, Germany).

Primary antibodies used were: chicken anti-TrkBex (1∶500; kindly provided by Dr. Reichardt at University of California, San Francisco); rabbit anti-p75NTR (1∶2000; Millipore; Billerica, MA); guinea pig anti-vesicular glutamate transporter 1 (vGluT1) (1∶1000; Synaptic Systems GmbH, Goettingen, Germany); rabbit anti-GFP (1∶1000; Invitrogen). Secondary antibodies used were: Alexa Fluor 488-conjugated goat anti-mouse IgG antibody; Alexa Fluor 555-conjugated goat anti-chicken IgG antibody; Alexa Fluor 488 or 555-conjugated goat anti-rabbit IgG antibody (Invitrogen); Cy3-conjugated donkey anti-guinea pig IgG antibody (Jackson ImmunoResearch Laboratories; West Grove, PA). All secondary antibodies were used with 1∶500 dilution.

### Western blot analysis

The olfactory bulbs from 5 day old mice were homogenized in TNE buffer [150 mM NaCl, 10 mM Tris-HCl (pH 7.4), 5 mM EDTA] containing protease inhibitors cocktail. The protein concentration in the homogenate was determined using Bradford reagent (Thermo Fisher Scientific; Rockford, IL). The sample (25 µg protein/lane) was subjected to 1D-SDS-PAGE (7.5%) and transferred onto a nitrocellulose membrane (Bio-Rad Laboratories, Hercules, CA).

Membrane was placed into blocking buffer [150 mM NaCl, 10 mM Tris-HCl (pH 7.4), 5% skim milk with 0.05% Tween-20] for 60 minutes at room temperature and incubated at 4°C overnight with anti-TrkBex antibody (1∶2000) diluted in the blocking buffer. The membrane was washed with blocking buffer and further incubated with blocking buffer containing chicken-specific secondary antibody conjugated to horseradish peroxidase (1∶10000; Jackson ImmunoResearch Laboratories) for 60 minutes at room temperature. The membrane was washed, and followed by enhanced chemiluminescence.

### Primary culture

Mouse pups at P1 were rapidly decapitated and the OB was dissected out in cold sterile Hanks' Balanced Salt Solution (HBSS). After a brief centrifugation (1,500 rpm) the tissue was enzymatically digested for 1 hour using the Papain Dissociation System (Worthington Biochemical Corporation; Lakewood, NJ), according to manufacturer instructions. Dissociated cells were resuspended in Neurobasal Medium supplemented with B-27, 100 units/ml Penicillin/Streptomycin, and 0.5–2 mM L-Glutamine (Invitrogen), and 17,500 cells were plated on 8-well chambered slides (BD Biosciences; San Jose, CA) previously coated overnight with 100 µg/ml of Poly-D-Lysine (Sigma; St. Louis, MO). After 3 hours of incubation at 37°C in a 5% CO_2_ atmosphere, recombinant human neurotrophins (BDNF, NT3, or NT4, PeproTech Inc., Rocky Hill, NJ; 1 or 10 ng/ml) with/without recombinant human TrkBFc chimera proteins (R&D Systems; Minneapolis, MN; 1 µg/ml) were added to the medium. Cells were fixed at 4 DIV.

For immunocytochemistry, cultured cells were first fixed with 2% PFA/2% sucrose in culture medium for 15 minutes at room temperature, followed by fixation with 4% PFA/4% sucrose in PBS for another 15 minutes, and washed with PBS. Fixed neurons were permeabilized with PBS-T (PBS with 0.25% Triton-X) for 5 minutes, washed with PBS, blocked with 3% BSA/PBS for 60 minutes, and incubated for 3 hours at room temperature with primary antibodies diluted in 3% BSA/PBS. Neurons were washed with PBS, incubated for 60 minutes with secondary antibodies and DAPI at room temperature, washed with PBS, and mounted with Gel/Mount.

### Intracellular dye injection

For intracellular dye injection, wild-type mice at P3, 5, 7, 10 and 28 were transcardially perfused with either of two fixatives (4% PFA or 4% PFA/0.125% glutaraldehyde), followed by postfixation with 4% PFA for 5–7 hours. The OBs were coronally cut on a vibratome (Ted Pella, Inc; Redding, CA) into 300 µm slices, and stored in 0.1 M PB (pH 7.4) at 4°C until use.

At the time of injection, 6% Lucifer yellow (Invitrogen) diluted with 50 mM Tris-HCl (pH 7.4) was filled into a glass micropipette (impedance 100–200 MΩ). The slices were mounted onto a membrane filter (Millipore) and transferred into an injection chamber containing 0.1 M PB (pH 7.4). Under the fluorescent microscope, the micropipette was placed in the MCL, and Lucifer yellow was injected with a negative current (1–10 nA) for 10–20 minutes until the glomerular tufts of the mitral cell appeared brightly fluorescent. Lucifer yellow-injected slices were then post-fixed with 4% PFA for at least 48 hours at 4°C and cut on a vibratome into 50 µm sections. Sections were sequentially incubated with blocking buffer (2% BSA in PBS-T), rabbit anti-Lucifer yellow antibody (Invitrogen; 1∶200), and Alexa Fluor 555-conjugated goat anti-rabbit IgG antibody with DAPI and DRAQ5.

### Image acquisition and statistics

To analyze YFP-positive cells in primary culture, images were acquired using an epifluorescent microscope (BX51, Olympus Corporation; Tokyo, Japan) with 10×or 60×objectives. Levels were adjusted in Photoshop software (Adobe; San Jose, CA), but the images were otherwise unaltered. Neurite analysis (numbers of branching points and primary neurites, total neurite length, and maximum neurite length) of YFP-positive cells and counting of DAPI and YFP-positive cells were performed with images taken with 10×objective, whose minimum resolution is 1.3 µm (Fig. S2A2). Neurites were traced (Fig. S2A1) and numbers of branching points and neurites extending from cell body (primary neurites), length of the total traced neurites (total neurite length), and maximum neurite length were calculated using HCA-Vision software (CSIRO; NSW, Australia). At least 76 YFP-positive cells having neurites longer than 10 µm were analyzed for every condition and three independent repetitions were done. Measurement of filopodia was performed using Imaris software (Bitplane AG; Zurich, Switzerland). Images taken with 60×objective were used and thin and short protrusions extending from thicker neurites were defined as filopodia (Fig. S2A3). Differences between control and neurotrophin with/without TrkBFc treated cells were analyzed by unpaired *t* test using GraphPad Prism 4 software (GraphPad Software; San Diego, CA).

To analyze dendritic structure of Lucifer yellow-labeled mitral cells, images were acquired with a laser scanning confocal microscope using 40×or 63×oil-immersion objectives, and up to 2×digital zoom. *Z*-stacks were taken through the area of interest, 0.5 µm steps between images. Morphologies of labeled cells were reconstituted with Photoshop software. Total length and number of branching points of dendritic tufts in a glomerulus were analyzed using Neurolucida software (MBF Bioscience; Williston, VT).

## Supporting Information

Figure S1 Dendritic morphology of mitral cells projecting into multiple glomeruli. A, B: Lucifer yellow-labeled mitral cell at P5 (A) and P7 (B). Representative morphologies of apical dendrites projecting into two glomeruli are shown. Scale bars: 20 µm.
(0.09 MB TIF)

Figure S2 Morphological analysis of YFP-positive cells in culture. A: A YFP-positive cell cultured for 4 days in control condition. Image taken with 10×objective (A2) was traced with HCA-Vision software (A1), and total neurite length, numbers of primary neurites and branching points, and maximum neurite length were analyzed. Branching points are indicated with arrowheads. A neurite of the cell shown in A2 (square) was imaged with 60×objective (A3). Note that filopodia seen in A3 (asterisks) were not traced in A1. Scale bars: 10 µm.
(0.57 MB TIF)
