# Supplementary figures and images for: Dendritic Branching of Olfactory Bulb Mitral and Tufted Cells: Regulation by TrkB
Source: PLoS One. 2009 Aug 25;4(8):e6729. doi: 10.1371/journal.pone.0006729 (PMC2727791; doi:10.1371/journal.pone.0006729)

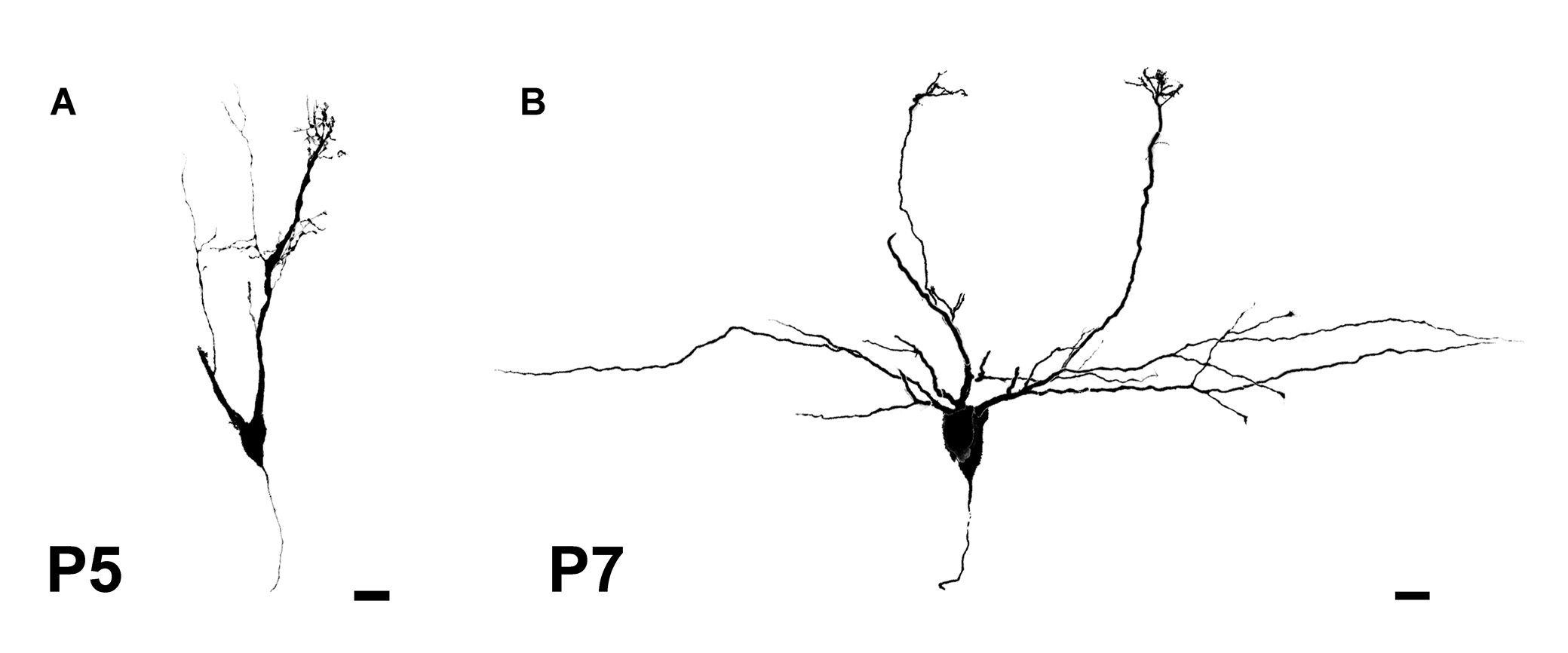

Supplement: Figure S1 — Dendritic morphology of mitral cells projecting into multiple glomeruli. A, B: Lucifer yellow-labeled mitral cell at P5 (A) and P7 (B). Representative morphologies of apical dendrites projecting into two glomeruli are shown. Scale bars: 20 µm. (0.09 MB TIF) [file pone.0006729.s001.tif]

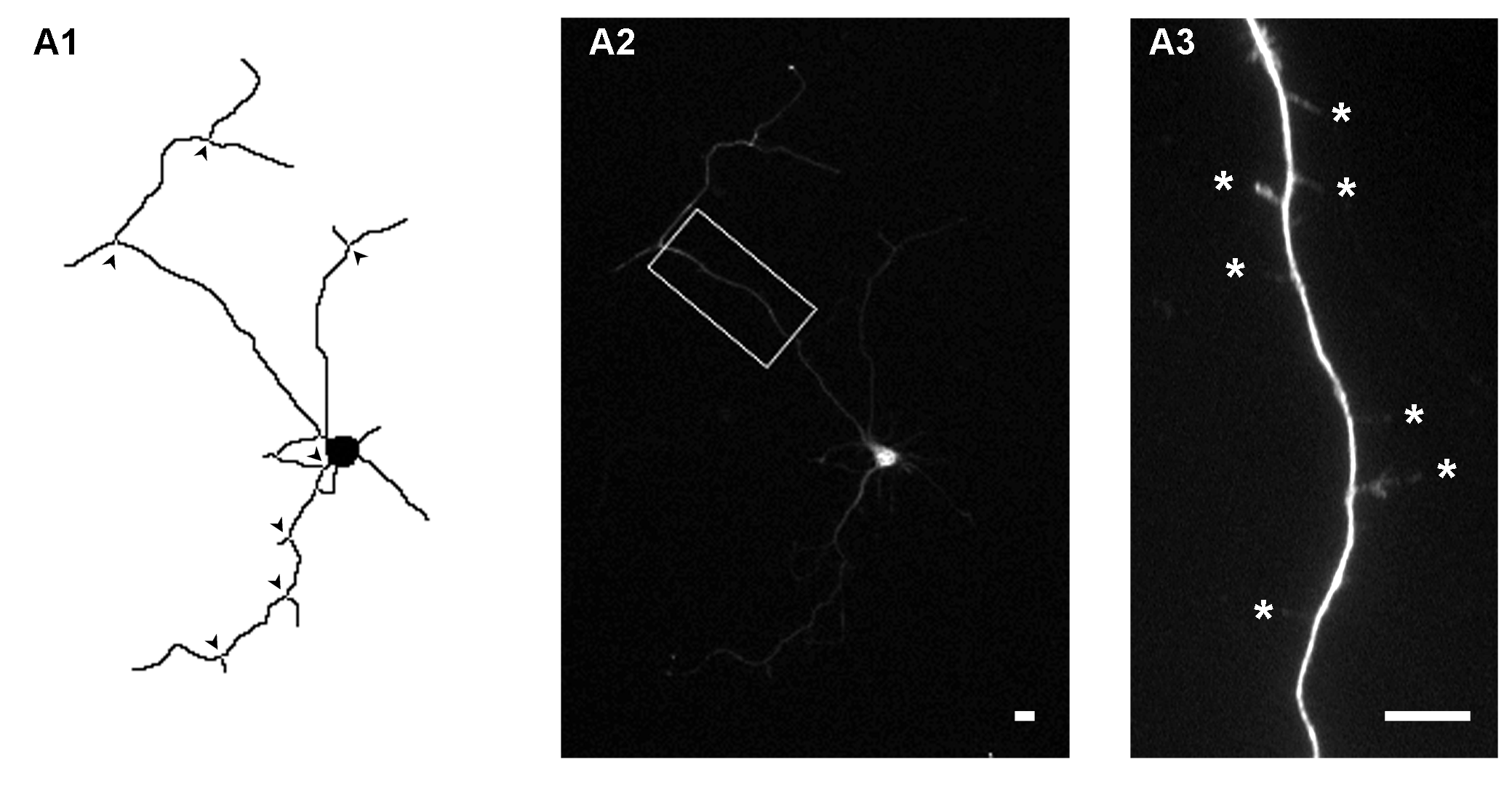

Supplement: Figure S2 — Morphological analysis of YFP-positive cells in culture. A: A YFP-positive cell cultured for 4 days in control condition. Image taken with 10×objective (A2) was traced with HCA-Vision software (A1), and total neurite length, numbers of primary neurites and branching points, and maximum neurite length were analyzed. Branching points are indicated with arrowheads. A neurite of the cell shown in A2 (square) was imaged with 60×objective (A3). Note that filopodia seen in A3 (asterisks) were not traced in A1. Scale bars: 10 µm. (0.57 MB TIF) [file pone.0006729.s002.tif]
